# *catena*-Poly[[bis­(acetato-κ^2^ *O*,*O*′)cobalt(II)]-μ-4,4′-bis­(benzimidazol-1-yl)biphenyl-κ^2^ *N* ^3^:*N* ^3′^]

**DOI:** 10.1107/S160053681004715X

**Published:** 2010-11-24

**Authors:** Ping-Yun Huang, Jin-Guo Wang

**Affiliations:** a College of Science, Chang’an University, Xi’an 710064, Shaanxi, People’s Republic of China

## Abstract

In the title one-dimensional coordination polymer, [Co(C_2_H_3_O_2_)_2_(C_26_H_18_N_4_)]_*n*_, the Co^II^ atom (site symmetry 2) is coordinated by two *O*,*O*′-bidentate acetate ions and two 4,4′-bis­(benzimidazol-1-yl)biphenyl ligands in a distorted *cis*-CoN_2_O_4_ octa­hedral geometry. The bridging ligand, which is completed by crystallographic twofold symmetry, links the Co^II^ atoms into [10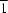] chains. Within the ligand, the dihedral angle between the benzene and benzimidazole rings is 48.31 (8)°.

## Related literature

For background to benzimidazole-derived ligands in coordin­ation polymers, see: Jin *et al.* (2006 ▶); Li *et al.* (2010 ▶); Su *et al.* (2003 ▶).
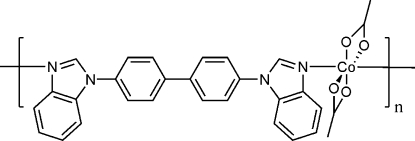


## Experimental

### 

#### Crystal data

[Co(C_2_H_3_O_2_)_2_(C_26_H_18_N_4_)]
*M* *_r_* = 563.46
Orthorhombic, 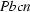

*a* = 13.078 (3) Å
*b* = 16.348 (3) Å
*c* = 11.354 (2) Å
*V* = 2427.5 (8) Å^3^
*Z* = 4
Mo *K*α radiation
μ = 0.75 mm^−1^
*T* = 293 K
0.25 × 0.22 × 0.18 mm

#### Data collection

Rigaku CCD area-detector diffractometer
Absorption correction: multi-scan (*CrystalClear*; Rigaku/MSC, 2005 ▶) *T*_min_ = 0.828, *T*_max_ = 0.873
21778 measured reflections
2150 independent reflections
2101 reflections with *I* > 2σ(*I*)
*R*_int_ = 0.038

#### Refinement

*R*[*F* ^2^ > 2σ(*F* ^2^)] = 0.035
*wR*(*F* ^2^) = 0.083
*S* = 1.10
2150 reflections
178 parameters
H-atom parameters constrained
Δρ_max_ = 0.27 e Å^−3^
Δρ_min_ = −0.41 e Å^−3^

### 

Data collection: *CrystalClear* (Rigaku/MSC, 2005 ▶); cell refinement: *CrystalClear*; data reduction: *CrystalClear*; program(s) used to solve structure: *SHELXS97* (Sheldrick, 2008 ▶); program(s) used to refine structure: *SHELXL97* (Sheldrick, 2008 ▶); molecular graphics: *SHELXTL* (Sheldrick, 2008 ▶); software used to prepare material for publication: *SHELXTL*.

## Supplementary Material

Crystal structure: contains datablocks I, global. DOI: 10.1107/S160053681004715X/hb5708sup1.cif

Structure factors: contains datablocks I. DOI: 10.1107/S160053681004715X/hb5708Isup2.hkl

Additional supplementary materials:  crystallographic information; 3D view; checkCIF report

## Figures and Tables

**Table 1:** Selected bond lengths (Å)

Co1—O1	2.0386 (16)
Co1—O2	2.4163 (18)
Co1—N1	2.0709 (16)

**Fig. 1.**
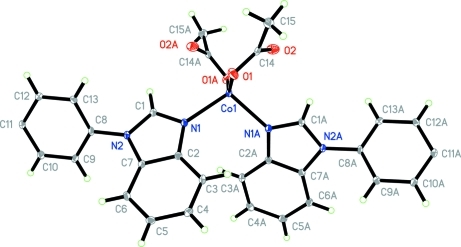
The molecular structure of (I). Displacement ellipsoids are drawn at the 30% probability level and H atoms are shown as small spheres of arbitrary radius.

**Fig. 2.**
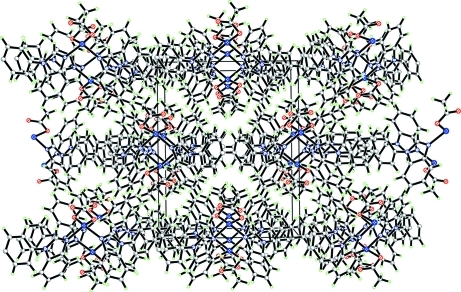
The crystal packing for (I).

## supplementary crystallographic information

### Comment

Benzoimidazole has been well used in crystal engineering, and a large number of benzoimidazole-containing flexible ligands have been extensively studied (Su *et al.*,2003; Jin *et al.*,2006). However, to our knowledge, the research on benzoimidazole ligands bearing rigid spacers is still less developed (Li *et al.*,2010).

Single-crystal X-ray diffraction analysis reveals that the title compound (I) crystallizes in the orthorhombic space group *Pbcn*. The geometry of the Co^II^ ion is surrounded by two benzoiimidazole rings of distinct L ligands and two chelated acetate anions, which illustrates a slightly distorted octahedral coordination environment (Fig. 1). Notably, as shown in Fig. 2, the six-coordinated Co^II^ center is bridged by the linear ligand L to form an infinite one-dimensional architecture.

### Experimental

A mixture of CH_3_OH and CHCl_3_ (1:1, 8 ml), as a buffer layer, was carefully layered over a solution of 4,4'-bis(benzoimidazol-1-yl)terphenyl (L, 0.06 mmol) in CHCl_3_ (6 ml). Then a solution of Co(CH_3_COO)_2_ (0.02 mmol) in CH_3_OH (6 ml) was layered over the buffer layer, and the resultant reaction was left to stand at room temperature. After *ca* three weeks, purple blocks of (I) appeared at the boundary. Yield: ~25% (based on L).

### Refinement

C-bound H atoms were positioned geometrically and refined in the riding-model approximation, with C—H = 0.93Å and *U*_iso_(H) = 1.2*U*_eq_.

### Crystal data

**Table:**

[Co(C_2_H_3_O_2_)_2_(C_26_H_18_N_4_)]	*D*_x_ = 1.542 Mg m^−^^3^
*M**_r_* = 563.46	Mo *K*α radiation, λ = 0.71073 Å
Orthorhombic, *P**b**c**n*	Cell parameters from 6002 reflections
*a* = 13.078 (3) Å	θ = 2.0–27.9°
*b* = 16.348 (3) Å	µ = 0.75 mm^−^^1^
*c* = 11.354 (2) Å	*T* = 293 K
*V* = 2427.5 (8) Å^3^	Block, purple
*Z* = 4	0.25 × 0.22 × 0.18 mm
*F*(000) = 1164	

### Data collection

**Table:**

Rigaku CCD area-detector diffractometer	2150 independent reflections
Radiation source: fine-focus sealed tube	2101 reflections with *I* > 2σ(*I*)
graphite	*R*_int_ = 0.038
Detector resolution: 9 pixels mm^-1^	θ_max_ = 25.0°, θ_min_ = 2.7°
ω scans	*h* = −15→15
Absorption correction: multi-scan (*CrystalClear*; Rigaku/MSC, 2005)	*k* = −19→19
*T*_min_ = 0.828, *T*_max_ = 0.873	*l* = −13→13
21778 measured reflections	

### Refinement

**Table:**

Refinement on *F*^2^	Primary atom site location: structure-invariant direct methods
Least-squares matrix: full	Secondary atom site location: difference Fourier map
*R*[*F*^2^ > 2σ(*F*^2^)] = 0.035	Hydrogen site location: inferred from neighbouring sites
*wR*(*F*^2^) = 0.083	H-atom parameters constrained
*S* = 1.10	*w* = 1/[σ^2^(*F*_o_^2^) + (0.0367*P*)^2^ + 2.7447*P*] where *P* = (*F*_o_^2^ + 2*F*_c_^2^)/3
2150 reflections	(Δ/σ)_max_ < 0.001
178 parameters	Δρ_max_ = 0.27 e Å^−^^3^
0 restraints	Δρ_min_ = −0.41 e Å^−^^3^

### Special details

**Table:**

Geometry. All e.s.d.'s (except the e.s.d. in the dihedral angle between two l.s. planes) are estimated using the full covariance matrix. The cell e.s.d.'s are taken into account individually in the estimation of e.s.d.'s in distances, angles and torsion angles; correlations between e.s.d.'s in cell parameters are only used when they are defined by crystal symmetry. An approximate (isotropic) treatment of cell e.s.d.'s is used for estimating e.s.d.'s involving l.s. planes.
Refinement. Refinement of *F*^2^ against ALL reflections. The weighted *R*-factor *wR* and goodness of fit *S* are based on *F*^2^, conventional *R*-factors *R* are based on *F*, with *F* set to zero for negative *F*^2^. The threshold expression of *F*^2^ > σ(*F*^2^) is used only for calculating *R*-factors(gt) *etc*. and is not relevant to the choice of reflections for refinement. *R*-factors based on *F*^2^ are statistically about twice as large as those based on *F*, and *R*- factors based on ALL data will be even larger.

### Fractional atomic coordinates and isotropic or equivalent isotropic displacement parameters (Å^2^)

**Table:**

	*x*	*y*	*z*	*U*_iso_*/*U*_eq_	
Co1	0.0000	0.40968 (2)	0.2500	0.01345 (14)	
O1	0.08394 (11)	0.37040 (10)	0.39033 (14)	0.0268 (4)	
C11	0.45996 (14)	0.51680 (12)	−0.20359 (17)	0.0138 (4)	
C12	0.44299 (15)	0.44856 (12)	−0.13277 (18)	0.0160 (4)	
H12	0.4800	0.4009	−0.1461	0.019*	
C7	0.23045 (15)	0.58691 (12)	0.15147 (17)	0.0134 (4)	
C3	0.11760 (15)	0.61095 (13)	0.31741 (17)	0.0163 (4)	
H3	0.0633	0.5956	0.3655	0.020*	
N2	0.24267 (12)	0.52419 (10)	0.06971 (14)	0.0140 (4)	
N1	0.11225 (12)	0.48650 (10)	0.18429 (14)	0.0148 (4)	
C10	0.40024 (15)	0.58655 (13)	−0.18495 (18)	0.0171 (4)	
H10	0.4096	0.6322	−0.2327	0.021*	
C13	0.37154 (15)	0.45080 (12)	−0.04256 (18)	0.0162 (4)	
H13	0.3618	0.4054	0.0055	0.019*	
C8	0.31481 (14)	0.52118 (12)	−0.02460 (17)	0.0144 (4)	
C9	0.32729 (15)	0.58878 (12)	−0.09655 (18)	0.0164 (4)	
H9	0.2872	0.6351	−0.0857	0.020*	
C14	0.02557 (15)	0.31459 (12)	0.42490 (18)	0.0163 (4)	
O2	−0.06065 (12)	0.30382 (10)	0.38106 (15)	0.0292 (4)	
C4	0.17056 (15)	0.68295 (13)	0.33635 (18)	0.0184 (4)	
H4	0.1517	0.7165	0.3989	0.022*	
C1	0.17101 (15)	0.46685 (12)	0.09472 (17)	0.0153 (4)	
H1	0.1641	0.4184	0.0526	0.018*	
C15	0.06315 (17)	0.25921 (14)	0.5229 (2)	0.0249 (5)	
H15A	0.0636	0.2889	0.5958	0.037*	
H15B	0.1312	0.2408	0.5052	0.037*	
H15C	0.0185	0.2128	0.5297	0.037*	
C2	0.14891 (15)	0.56223 (12)	0.22333 (17)	0.0140 (4)	
C6	0.28339 (15)	0.65947 (12)	0.16999 (17)	0.0162 (4)	
H6	0.3372	0.6753	0.1216	0.019*	
C5	0.25205 (16)	0.70673 (13)	0.26368 (18)	0.0176 (4)	
H5	0.2858	0.7557	0.2792	0.021*	

### Atomic displacement parameters (Å^2^)

**Table:**

	*U*^11^	*U*^22^	*U*^33^	*U*^12^	*U*^13^	*U*^23^
Co1	0.0112 (2)	0.0150 (2)	0.0141 (2)	0.000	0.00353 (14)	0.000
O1	0.0208 (8)	0.0286 (9)	0.0311 (9)	−0.0010 (7)	0.0001 (7)	0.0116 (7)
C11	0.0112 (9)	0.0182 (11)	0.0121 (9)	−0.0018 (8)	0.0014 (8)	−0.0006 (8)
C12	0.0148 (10)	0.0151 (10)	0.0182 (10)	0.0020 (8)	0.0026 (8)	−0.0013 (8)
C7	0.0132 (10)	0.0144 (10)	0.0126 (9)	0.0016 (7)	0.0007 (8)	0.0005 (8)
C3	0.0146 (10)	0.0214 (11)	0.0131 (10)	0.0008 (8)	0.0029 (8)	0.0006 (8)
N2	0.0134 (8)	0.0153 (8)	0.0133 (8)	−0.0009 (6)	0.0049 (7)	−0.0002 (7)
N1	0.0145 (8)	0.0154 (9)	0.0146 (8)	−0.0018 (7)	0.0037 (7)	−0.0016 (7)
C10	0.0173 (10)	0.0173 (11)	0.0167 (10)	0.0001 (8)	0.0041 (8)	0.0032 (8)
C13	0.0176 (10)	0.0144 (10)	0.0166 (10)	−0.0023 (8)	0.0035 (8)	0.0012 (8)
C8	0.0109 (9)	0.0189 (10)	0.0135 (10)	−0.0017 (8)	0.0042 (8)	−0.0012 (8)
C9	0.0141 (10)	0.0166 (10)	0.0184 (10)	0.0031 (8)	0.0036 (8)	0.0001 (8)
C14	0.0138 (10)	0.0187 (10)	0.0165 (10)	0.0023 (8)	0.0040 (8)	−0.0055 (8)
O2	0.0279 (9)	0.0226 (8)	0.0372 (9)	−0.0004 (7)	−0.0079 (8)	−0.0027 (7)
C4	0.0200 (11)	0.0197 (11)	0.0154 (10)	0.0025 (8)	0.0005 (8)	−0.0043 (8)
C1	0.0139 (10)	0.0159 (10)	0.0162 (10)	−0.0007 (8)	0.0038 (8)	−0.0009 (8)
C15	0.0237 (12)	0.0244 (12)	0.0265 (12)	0.0012 (9)	0.0015 (9)	0.0031 (10)
C2	0.0126 (9)	0.0151 (10)	0.0144 (10)	0.0010 (8)	0.0004 (8)	0.0007 (8)
C6	0.0129 (9)	0.0176 (10)	0.0180 (10)	−0.0015 (8)	0.0020 (8)	0.0019 (8)
C5	0.0162 (10)	0.0147 (10)	0.0220 (11)	−0.0014 (8)	−0.0022 (8)	−0.0016 (8)

### Geometric parameters (Å, °)

**Table:**

Co1—O1^i^	2.0386 (16)	N1—C1	1.315 (3)
Co1—O1	2.0386 (16)	N1—C2	1.400 (3)
Co1—O2	2.4163 (18)	C10—C9	1.385 (3)
Co1—O2^i^	2.4163 (18)	C10—H10	0.9300
Co1—N1	2.0709 (16)	C13—C8	1.384 (3)
Co1—N1^i^	2.0709 (16)	C13—H13	0.9300
O1—C14	1.253 (3)	C8—C9	1.384 (3)
C11—C12	1.393 (3)	C9—H9	0.9300
C11—C10	1.398 (3)	C14—O2	1.245 (3)
C11—C11^ii^	1.486 (4)	C14—C15	1.516 (3)
C12—C13	1.387 (3)	C4—C5	1.403 (3)
C12—H12	0.9300	C4—H4	0.9300
C7—C6	1.390 (3)	C1—H1	0.9300
C7—N2	1.392 (2)	C15—H15A	0.9600
C7—C2	1.402 (3)	C15—H15B	0.9600
C3—C4	1.383 (3)	C15—H15C	0.9600
C3—C2	1.394 (3)	C6—C5	1.377 (3)
C3—H3	0.9300	C6—H6	0.9300
N2—C1	1.356 (3)	C5—H5	0.9300
N2—C8	1.428 (2)		
			
O1^i^—Co1—O1	143.29 (10)	C9—C8—C13	120.90 (18)
O1^i^—Co1—N1	106.93 (7)	C9—C8—N2	119.55 (17)
O1—Co1—N1	95.21 (6)	C13—C8—N2	119.55 (17)
O1^i^—Co1—N1^i^	95.21 (6)	C8—C9—C10	119.21 (18)
O1—Co1—N1^i^	106.93 (7)	C8—C9—H9	120.4
N1—Co1—N1^i^	105.34 (9)	C10—C9—H9	120.4
C14—O1—Co1	98.41 (13)	O2—C14—O1	122.0 (2)
C12—C11—C10	118.47 (18)	O2—C14—C15	120.16 (19)
C12—C11—C11^ii^	121.45 (13)	O1—C14—C15	117.86 (18)
C10—C11—C11^ii^	120.07 (13)	C3—C4—C5	121.67 (19)
C13—C12—C11	120.84 (18)	C3—C4—H4	119.2
C13—C12—H12	119.6	C5—C4—H4	119.2
C11—C12—H12	119.6	N1—C1—N2	113.41 (18)
C6—C7—N2	132.25 (18)	N1—C1—H1	123.3
C6—C7—C2	122.45 (18)	N2—C1—H1	123.3
N2—C7—C2	105.27 (17)	C14—C15—H15A	109.5
C4—C3—C2	117.29 (18)	C14—C15—H15B	109.5
C4—C3—H3	121.4	H15A—C15—H15B	109.5
C2—C3—H3	121.4	C14—C15—H15C	109.5
C1—N2—C7	106.87 (16)	H15A—C15—H15C	109.5
C1—N2—C8	126.16 (17)	H15B—C15—H15C	109.5
C7—N2—C8	126.95 (16)	C3—C2—N1	130.35 (18)
C1—N1—C2	105.12 (16)	C3—C2—C7	120.32 (19)
C1—N1—Co1	123.02 (14)	N1—C2—C7	109.32 (17)
C2—N1—Co1	131.69 (13)	C5—C6—C7	116.59 (18)
C9—C10—C11	121.05 (19)	C5—C6—H6	121.7
C9—C10—H10	119.5	C7—C6—H6	121.7
C11—C10—H10	119.5	C6—C5—C4	121.67 (19)
C8—C13—C12	119.46 (18)	C6—C5—H5	119.2
C8—C13—H13	120.3	C4—C5—H5	119.2
C12—C13—H13	120.3		
			
O1^i^—Co1—O1—C14	−43.39 (12)	C13—C8—C9—C10	−2.3 (3)
N1—Co1—O1—C14	−171.08 (13)	N2—C8—C9—C10	177.49 (18)
N1^i^—Co1—O1—C14	81.14 (14)	C11—C10—C9—C8	1.1 (3)
C10—C11—C12—C13	−2.7 (3)	Co1—O1—C14—O2	−6.9 (2)
C11^ii^—C11—C12—C13	176.7 (2)	Co1—O1—C14—C15	172.84 (15)
C6—C7—N2—C1	178.2 (2)	C2—C3—C4—C5	0.5 (3)
C2—C7—N2—C1	0.0 (2)	C2—N1—C1—N2	−1.1 (2)
C6—C7—N2—C8	−3.3 (3)	Co1—N1—C1—N2	−176.94 (13)
C2—C7—N2—C8	178.53 (18)	C7—N2—C1—N1	0.7 (2)
O1^i^—Co1—N1—C1	−49.07 (17)	C8—N2—C1—N1	−177.81 (17)
O1—Co1—N1—C1	101.29 (16)	C4—C3—C2—N1	178.6 (2)
N1^i^—Co1—N1—C1	−149.55 (18)	C4—C3—C2—C7	−0.5 (3)
O1^i^—Co1—N1—C2	136.34 (17)	C1—N1—C2—C3	−178.1 (2)
O1—Co1—N1—C2	−73.29 (18)	Co1—N1—C2—C3	−2.8 (3)
N1^i^—Co1—N1—C2	35.86 (15)	C1—N1—C2—C7	1.1 (2)
C12—C11—C10—C9	1.4 (3)	Co1—N1—C2—C7	176.39 (13)
C11^ii^—C11—C10—C9	−178.0 (2)	C6—C7—C2—C3	0.2 (3)
C11—C12—C13—C8	1.5 (3)	N2—C7—C2—C3	178.57 (17)
C12—C13—C8—C9	1.0 (3)	C6—C7—C2—N1	−179.06 (18)
C12—C13—C8—N2	−178.74 (17)	N2—C7—C2—N1	−0.7 (2)
C1—N2—C8—C9	131.6 (2)	N2—C7—C6—C5	−177.7 (2)
C7—N2—C8—C9	−46.6 (3)	C2—C7—C6—C5	0.2 (3)
C1—N2—C8—C13	−48.6 (3)	C7—C6—C5—C4	−0.3 (3)
C7—N2—C8—C13	133.1 (2)	C3—C4—C5—C6	−0.1 (3)

Symmetry codes: (i) −*x*, *y*, −*z*+1/2; (ii) −*x*+1, *y*, −*z*−1/2.
